# When bad turns good: a systematic review on cholesterol and LDL in longitudinal patient cohorts with Parkinson’s disease

**DOI:** 10.1007/s10072-026-09070-9

**Published:** 2026-05-13

**Authors:** Rea Lumi, Julia Tartakovskaja, Lan Ye, Berit Abraham, Clara Niesmann, Stephan Greten, Florian Wegner, Martin Klietz

**Affiliations:** Department of Neurology, Hannover Medical School, Carl-Neuberg-Straße, 1, 30625 Hannover, Germany

**Keywords:** Parkinson’s disease, Cholesterol, Low-density lipoprotein cholesterol, Pathogenesis, Biomarker

## Abstract

**Background:**

While the aetiology of Parkinson’s disease (PD) involves genetic and environmental factors, emerging evidence has suggested a surprising link between lipid metabolism - particularly serum total cholesterol (TC) and low density lipoprotein cholesterol (LDL-C) - and the pathogenesis of PD. Cholesterol plays a vital role in neuronal membrane integrity, myelination, and synaptic function. However, its peripheral concentration and regulatory mechanisms in the central nervous system (CNS), remain incompletely understood in the context of PD.

**Objective:**

This review aims to systematically examine the current literature on the relationship between TC and LDL-C with the risk and progression of PD in longitudinal patient cohorts.

**Methods:**

A systematic literature search was conducted using PubMed with the keywords “Parkinson’s disease” AND “cholesterol” and “Parkinson’s disease” AND “LDL.” Inclusion criteria encompassed English-language longitudinal human studies published before October 2024, with data on cholesterol levels in relation to PD incidence or progression. Fourteen studies met the eligibility criteria.

**Results:**

Twelve of the 14 included studies reported an inverse association between lower serum total cholesterol (TC) and low-density lipoprotein cholesterol (LDL-C) levels and increased risk for PD onset as well as faster disease progression. Notably, this pattern was not uniform across all subgroups: age, sex, BMI, and statin use modulated the strength and direction of associations.

**Conclusion:**

Despite some heterogeneity across studies, there is growing evidence that lower TC and LDL-C may be associated with a higher risk and more rapid progression of PD. Future research should focus on mechanistic studies and stratified analyses to clarify whether and how cholesterol modulation could contribute to neuroprotective strategies in PD.

**Supplementary Information:**

The online version contains supplementary material available at 10.1007/s10072-026-09070-9.

## Introduction

Parkinson’s disease (PD) is the second most common neurodegenerative disorder after Alzheimer’s disease [1], affecting approximately 1.51 per 1,000 individuals worldwide and increases in prevalence with age, particularly among older men [2].

PD is defined by the progressive loss of dopaminergic neurons in the substantia nigra and by the accumulation of aggregated α-synuclein within Lewy bodies, which gives rise to the hallmark motor symptoms tremor, rigidity, bradykinesia and postural instability [3, 4]. Non-motor symptoms such as depression, cognitive impairment and sleep disorders are also common and significantly impact quality of life [5].

Multimorbidity is highly prevalent in PD and was shown to accelerate functional decline and worsen outcomes [6]. For instance, comorbid diabetes mellitus and osteoarthritis are independently associated with faster progression of motor disability and reduced mobility in PD patients [7]. Such observations underscore the importance of exploring metabolic factors beyond classical risk markers.

Cholesterol is an essential lipid found in all human cells, serving as a key structural component of cell membranes and a precursor for the synthesis of steroid hormones and vitamin D [8, 9]. It is either ingested through animal-based foods or synthesized endogenously in the liver via the mevalonate pathway, with tightly regulated feedback mechanisms responding to metabolic demand [10].

Roughly 70–80% of central nervous system (CNS) cholesterol is embedded in myelin sheaths; this arrangement electrically insulates neurons and supports high-speed signal conduction [11, 12]. Additionally, cholesterol is the precursor for bile acids required for lipid digestion [13]. Although serum cholesterol itself cannot cross into the brain, peripheral lipid metabolism may influence central processes via oxysterols and lipoprotein-mediated signaling [14].

Total cholesterol (TC) includes several lipoprotein-bound fractions such as low-density lipoprotein (LDL), high-density lipoprotein (HDL), and very low-density lipoprotein (VLDL). LDL, often referred to as “bad” cholesterol, is a protein-fat complex that transports cholesterol from the liver to peripheral tissues, where elevated levels promote disposition in arterial walls (atherogenesis), leading to plaque formation and significantly increasing the risk for cardiovascular diseases (CVD) such as heart attack, stroke and peripheral artery disease [15, 16].

HDL returns excess cholesterol to the liver, while VLDL primarily carries triglycerides. Clinically, elevated LDL and low HDL levels are associated with increased cardiovascular risk [17]. In this overview, LDL is prioritized because it is a most well-established risk factor for atherosclerosis – unlike HDL (which acts protectively), triglycerides (which are secondarily linked to metabolic syndrome but less directly atherogenic), or Lipoprotein a [Lp(a)] (a genetically determined factor that is supplementary but not primarily therapeutically targetable). Targeted LDL reduction through lifestyle measures or statins thus remains the cornerstone of cardiovascular prevention.

Building on this biochemical background, studies investigating the relationship between serum total cholesterol, LDL-C levels and PD have yielded conflicting findings: while many reports found an increased incidence of PD in individuals with lower cholesterol, a few implicate higher cholesterol as a potential risk factor [18, 19].

However, understanding whether cholesterol acts as a biomarker or modifiable risk factor in PD could inform both preventive strategies and therapeutic interventions.

This review aims to provide current evidence from patient cohorts regarding the associations between serum total cholesterol, LDL-C and both the incidence and progression of PD, with the intention of offering insights into the potential role in PD pathophysiology and supporting future clinical research.

## Methods

This review is based on a systematic search of the scientific literature to identify studies that examine the association between TC and LDL-C with the incidence and progression of PD. The search was conducted exclusively using the PubMed database. The systematic review was performed in accordance with the PRISMA 2020 guidelines. Only English-language publications were included.

To retrieve relevant studies, the following search terms were used: “Parkinson’s disease” *AND* “cholesterol” and “Parkinson’s disease” AND “LDL”. All studies selected for review were identified through these specific search terms. The resulting publications were first screened for relevance by reviewing their titles and abstracts. Duplicate entries were manually excluded. Full texts were then assessed to determine eligibility based on pre-established inclusion and exclusion criteria.

**Fig. 1:**
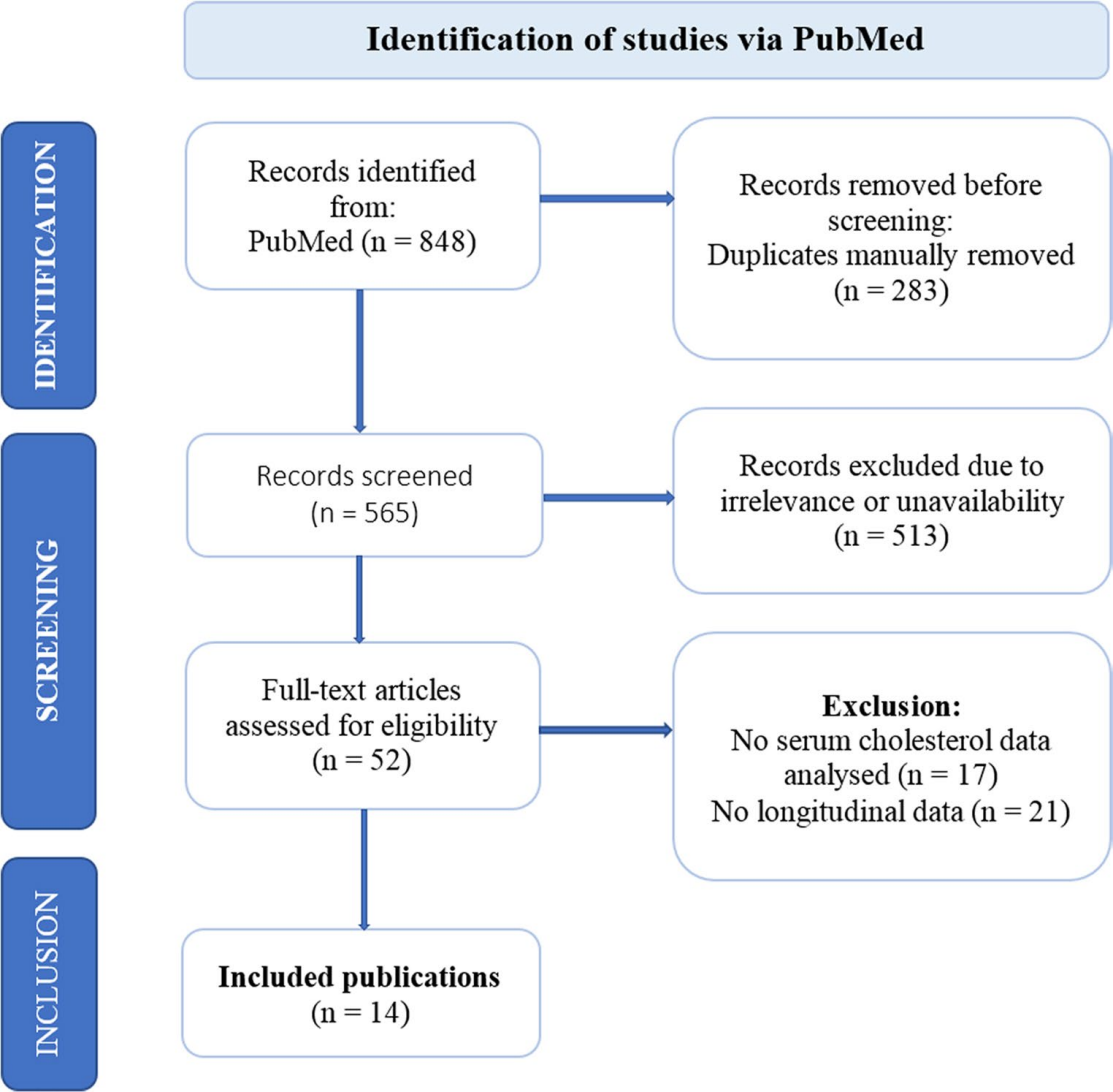
Flow diagram of the literature search process, own representation based on PRISMA 2020 Flow diagram for new systematic reviews

Studies were included if they had been published prior to October 1, 2024, contained longitudinal data on individuals with PD (with or without control groups), and investigated the relationship between TC and/or LDL-C levels and either the onset or the progression of PD. Studies that lacked longitudinal data, as well as review articles, meta-analyses, and animal-based research, were excluded from the analysis. The overall process of study selection is illustrated in Fig. 1.

All included studies provided longitudinal data with follow-up durations ranging from 3 years [20, 21] to observation periods of up to 20 years before diagnosis [19].

The quality of the included studies was independently assessed by two authors (R.L. and M.K.) using the Newcastle-Ottawa Scale (NOS) for cohort studies [22]. Any disagreements were resolved by consensus with a third author (F.W.). The NOS evaluates three domains: selection of study groups (maximum 4 stars), comparability of groups (maximum 2 stars), and ascertainment of outcome (maximum 3 stars), with a maximum total of 9 stars. Studies scoring 7–9 stars were considered to be of good quality.

## Results

A total of 14 studies met the inclusion criteria. Eight of these investigated the relationship between serum cholesterol levels and the risk of developing PD, while six focused on disease progression. The findings are presented below, categorized according to these two aspects (Tables 1 and 2).

**Table 1:** Association between blood lipid levels and risk of Parkinson’s disease across different population-based cohorts (incidence analysis)

Nr.	References	Country	Sample size	Study design	HR / OR / RR (95% CI)	*p*-Value
1.	De Lau et al. [23]	Netherlands	6,465	Population-based cohort	HR 0.77 per mmol/L (women only) (0.64–0.94)	0.002
2.	Simon et al. [24]	United States	112,000	Prospective cohort (self-report)	RR 0.86 per 50 mg/dL TC (0.78–0.95)	0.07
3.	Hu et al. [18]	Finnland	50,926	Population-based cohort	HR increased with TC (exact value not mentioned)	0.002
4.	Huang et al. [25]	United States (Hawaii)	3,233	Elderly male cohort	OR 0.40 (0.20–0.90) for highest vs. lowest LDL-C	0.03
5.	Huang et al. [26]	United States	15,792	Prospective cohort	OR 0.43 (0.22–0.87) for highest vs. lowest TC tertile	0.02
6.	Rozani et al. [27]	Israel	261,638	Prospective cohort	TC: HR 0.71 (0.55–0.93); LDL-C: HR 0.72 (0.54–0.95)	< 0.05
7.	Fang et al. [19]	Sweden	> 600,000	Retrospective cohort	TC: HR 0.90 (0.87–0.94); LDL-C: HR 0.93 (0.88–0.98)	0.001, 0.02
8.	Hurh et al. [28]	South Korea	200,454	Population-based cohort	TC: HR 1.17 (1.04–1.31); LDL-C: HR 1.19 (1.06–1.34)	0.05

Abbreviations: *TC* - Total Cholesterol; *LDL-C* - Low-Density Lipoprotein Cholesterol; *PD* - Parkinson’s Disease; *HR* - Hazard Ratio; *OR* - Odds Ratio; *RR* - Relative Risk; *CI* - Confidence Interval; *p-value* - Probability Value

**Table 2:** Association between blood lipid levels and risk of Parkinson’s disease progression (longitudinal analysis)

Nr.	References	Country	Sample size	Study design	Main Outcome / Association	HR / CIR / OR / β (95% CI)	*p*-Value
1.	Huang et al. [29]	United States	800 PD patients (early stage)	RCT cohort (DATATOP study)	Higher TC associated with slower progression (borderline significance in men)	HR = 0.88 (0.77–1.00) [men]	0.05
2.	Sterling et al. [20]	United States	64 PD patients, 64 controls	Longitudinal observational	Higher LDL-C associated with better executive and motor function in PD	β = 0.003 (exec.), β = 0.002 (motor)	< 0.001; 0.030
3.	Huang et al. [21]	United States	60 PD patients, 64 controls (matched cohort)	Case-control with 3y follow-up	(S)24-OH-cholesterol inversely associated with PD risk and positively with olfaction	OR = 0.92 (0.87–0.97)	0.004
4.	Yokoi et al. [30]	Japan	45 PD patients, 120 controls	Case-control	Lower TC/LDL-C in PD vs. controls; sex- and time-dependent lipid decline	-	-
5.	Wang et al. [14]	United States	3,053 older adults (70–79 y)	Prospective cohort (Health ABC)	Lower TC associated with higher PD progression risk	CIR 0.41 (0.20–0.86); CIR 0.69 (0.35–1.35)	-
6.	Jeong et al. [31]	South Korea	321 PD patients	Observational, BMI-stratified	TC linked to better cognition in normal weight, worse in obese	β = 0.205 / -0.213	0.013 / 0.017

Abbreviations: *PD* - Parkinson’s Disease; *TC* - Total Cholesterol; *LDL-C* - Low-Density Lipoprotein Cholesterol; *HR* - Hazard Ratio; *OR* - Odds Ratio; *β* - Beta coefficient (effect size in regression); *CI* - Confidence Interval; *RCT* - Randomized Controlled Trial; *CIR* - cumulative incidence ratio (accounting for competing risk of death); *p-value* - Probability Value

### Association between serum cholesterol and PD-onset

Seven of the eight studies that examined PD incidence reported a significant inverse association between lower TC and/or LDL-C levels and higher risk of developing PD. Hurh et al. analysed data from 200,454 individuals in South Korea and found that lower levels of TC and LDL-C were associated with a significantly increased risk of developing PD. Participants in the lowest TC tertile had a 1.17-fold higher risk (HR 1.17; 95% CI 1.04–1.31, *p* < 0.05) and those in the lowest LDL-C tertile had a 1.19-fold increased risk (HR 1.19; 95% CI 1.06–1.34; *p* < 0.05), each compared with those in the middle tertile [28].

Similarly, Fang et al., using data from over 600,000 individuals in Sweden, reported that higher levels of TC and LDL-C were associated with a lower risk of PD. A one standard deviation increase in TC reduced the risk by 10% (HR 0.90; 95% CI 0.87–0.94; *p* < 0.001) and a similar increase in LDL-C was linked to a 7% risk reduction (HR 0.93; 95% CI 0.88–0.98; *p* = 0.02), respectively [19]. Notably, lipid differences were detectable up to 20 years before diagnosis.

Rozani et al. examined 261,638 individuals in Israel and observed that men with higher TC (> 180 mg/dL) and LDL-C (> 110 mg/dL) had a significantly lower risk of developing PD, with hazard ratios of 0.71 (95% CI 0.55–0.93; *p* < 0.05) and 0.72 (95% CI 0.54–0.95; *p* < 0.05), respectively [27]. Among women, a protective effect was found only in those aged 70–74 years.

De Lau et al. evaluated 6,465 participants from the Rotterdam Study. Their analysis revealed a sex-specific association: in women, higher TC levels were linked to a reduced PD risk (HR 0.77 per mmol/L increase; 95% CI 0.64–0.94; *p* for trend = 0.002), while no significant effect was observed in men [23].

Huang et al. found in a U.S. cohort of 15,792 individuals that use of statins before 1998 was associated with increased PD risk. After adjusting for statin intake, higher TC remained inversely associated with PD (OR 0.43 for highest vs. lowest tertile; 95% CI 0.22–0.87; *p* = 0.02), with similar findings for LDL-C [26].

In a cohort of 3,233 elderly Japanese-American men, Huang et al. reported a significant reduction in PD incidence with higher LDL-C levels, particularly in men aged 71–75. The odds ratio for those in the highest LDL-C percentile versus the lowest was 0.40 (95% CI 0.2–0.9; *p* = 0.03) [25].

Hu et al. evaluated 50,926 Finnish individuals and found that higher TC levels were associated with an increased risk of PD, particularly in the 25–54 age group. Hazard ratios increased consistently across cholesterol categories (*p* for trend = 0.002) [18].

Simon et al., calculating data from over 112,000 U.S. health professionals, reported a slight but statistically non-significant inversed association between TC levels and the risk of PD (RR 0.86 per 50 mg/dL; 95% CI 0.78–0.95; *p* = 0.07) [24].

### Association between serum cholesterol and Parkinson’s disease progression or clinical features

Five of the six studies that investigated disease progression or related clinical features found that higher TC and LDL-C levels were associated with slower motor and cognitive decline or better preservation of specific functions.

Wang et al. analysed data from the Health ABC Study involving 3,053 U.S. adults aged 70–79. Participants were divided into tertiles based on their TC levels, meaning the entire cohort was split into three equally sized groups: the lowest third (lowest TC values; mean TC ~ 162.6 mg/dL), the middle third (mean TC ~ 200.4 mg/dL), and the highest third (mean TC ~ 244.9 mg/dL). Lower baseline TC was linked to higher PD risk.

Cholesterol levels began to decrease in the prodromal stage of PD, becoming significantly lower approximately 4 years before disease diagnosis (observed-expected difference: -6.68 mg/dL; 95% CI: -13.14, − 0.22) and further declined by year 6 post-diagnosis (-13.59 mg/dL; 95% CI: -22.12, -5.06), though the linear trend was not statistically significant (*p* = 0.10). PD incidence was lower in the second (CIR = 0.41; 95% CI: 0.20–0.86) and third (CIR = 0.69; 95% CI: 0.35–1.35) TC tertiles compared to the lowest, with significance only in the second tertile [14].

Yokoi et al. (2020) examined 45 PD patients (22 men, 23 women) and 120 matched controls in Japan and found that both male and female PD patients had significantly lower baseline TC levels compared to controls (men: 190.7 ± 33.2 vs. 212.4 ± 21.9 mg/dL, *p* = 0.001; women: 216.2 ± 26.9 vs. 234.9 ± 30.6 mg/dL, *p* = 0.012). LDL-C levels were significantly lower in male PD patients (107.8 ± 25.9 vs. 122.9 ± 24.2 mg/dL, *p* = 0.015), but not in women (122.6 ± 25.3 vs. 132.1 ± 25.0 mg/dL, *p* = 0.131). In men, cholesterol levels declined before the onset of motor symptoms (5 to 1 years prior) and continued to decrease during disease progression, whereas in women, lipid levels decreased only after symptom onset. This sex-specific pattern of lipid reduction relative to disease course was quantitatively confirmed [30]. Given the small sample size, these findings warrant cautious interpretation.

Sterling et al. followed 64 PD patients and 64 controls for three years. Within the PD group, higher LDL-C levels were significantly associated with better executive function (β = 0.003, *p* < 0.001) and fine motor skills (β = 0.002, *p* = 0.030) over time [20]. No such associations were found in the control group, suggesting that higher LDL-C levels may play a protective role in the progression of PD.

In the DATATOP study, Huang et al. assessed 800 early-stage PD patients who had not yet started dopaminergic therapy. Participants with higher TC showed a slower clinical progression toward requiring dopaminergic medication (HR per SD = 0.90; 95% CI 0.80–1.01). While the overall trend was not statistically significant, it reached borderline significance in men (HR = 0.88; 95% CI 0.77-1.00; *p* = 0.05), but not in women [29].

Jeong et al. analysed 321 South Korean PD patients grouped by body mass index (BMI). Higher TC was positively associated with better cognitive function (specifically frontal executive ability) in normal-weight patients (β = 0.205, *p* = 0.013), but negatively associated in obese individuals (β = −0.213, *p* = 0.017). Cox regression analyses showed opposing risks of dementia depending on BMI group [31].

Huang et al. conducted a three-year follow-up study comparing 60 PD patients with 64 matched controls. Although general serum cholesterol levels were lower in the PD group, only (S)24-OH-cholesterol (a brain-derived metabolite) was significantly associated with PD risk (OR per 1 ng/mL = 0.92; 95% CI 0.87–0.97; *p* = 0.004). Higher levels of (S)24-OH were also linked to better olfactory function, while no relevant associations were found with peripheral cholesterol metabolites such as 27-OH [21].

## Discussion

Overall, this systematic review underlines a consistent inverse association between peripheral cholesterol/LDL-C levels and the incidence/progression of PD. Several large epidemiological cohorts indicate that lower peripheral cholesterol levels are linked to increased PD risk and faster disease progression [14, 21, 23]. In particular, data from the Rotterdam Study and the Honolulu-Asia Aging Study highlight a potential protective window in the “early-seventies”, where higher TC and LDL-C levels correlated with reduced PD incidence and slower motor decline [14, 25]. These findings suggest a need for further research into lipid management in the context of neurodegeneration, while acknowledging the established cardiovascular risks of elevated lipids.

The majority of studies were rated as good quality (7–9 stars on the NOS), indicating low risk of bias in selection, comparability and outcome assessment. Comparability was occasionally limited due to incomplete adjustment for important confounders such as statin use or BMI (Supplementary Table 1).

Several important confounders may influence the observed association between serum cholesterol/LDL-C levels and PD risk or progression. Statin use was assessed and adjusted for in the majority of studies; however, differentiation between lipophilic and hydrophilic statins was rarely performed. Dietary patterns and adherence to a Mediterranean-style diet were only inconsistently reported. BMI was recorded in most cohorts and showed differential effects (Jeong et al.), with higher cholesterol appearing protective in normal-weight but potentially detrimental in obese patients. To enhance transparency, we summarised the assessment of these confounders across all included studies in Table 3. Although many studies attempted statistical adjustment, residual confounding cannot be entirely excluded and may partly explain the observed heterogeneity. In addition, substantial heterogeneity in study populations (age range, sex distribution, ethnic background, and geographic origin) likely contributed to some of the observed variability in effect sizes and association direction.

**Table 3:** Assessment of major confounders (statin use, diet, BMI and other lifestyle factors) in the included studies

Nr.	Study	Statin use assessed/adjusted	Dietary information	BMI assessed	Other lifestyle factors
1.	De Lau et al. (2006)	Not reported	Not reported	Yes	Age, sex
2.	Simon et al. (2007)	Yes	Not reported	Yes	Age, sex, smoking, physical activity
3.	Huang et al. (2008)	Not reported	Not reported	Yes	Age, sex
4.	Hu et al. (2008)	Not reported	Not reported	Yes	Age, sex, smoking, alcohol
5.	Huang et al. (2011)	Yes	Not reported	Yes	Age, sex
6.	Huang et al. (2015)	Yes	Not reported	Yes	Age, sex, smoking
7.	Sterling et al. (2016)	Yes	Not reported	Yes	Age, sex, education
8.	Rozani et al. (2018)	Yes (Statin-free-cohort)	Not reported	Yes	Age, sex
9.	Fang et al. (2019)	Yes	Not reported	Yes	Age, sex, smoking, alcohol
10.	Huang et al. (2019)	Yes	Not reported	Yes	Age, sex
11.	Yokoi et al. (2020)	Yes	Not reported	Yes	Age, sex
12.	Wang et al. (2021)	Yes	Not reported	Yes	Age, sex, comorbities
13.	Hurh et al. (2022)	Yes	Not reported	Yes	Age, sex, comorbities
14.	Jeong et al. (2024)	Yes	Not reported	Yes (stratified)	Age, sex, disease duration

Note: Information on confounders was extracted from the Methods sections of the original publications. “Not reported” means that the respective information (especially dietary assessment) was not described in the study

A central question that remains is whether reduced peripheral cholesterol levels are a causal contributor to PD pathogenesis or rather a consequence of the neurodegenerative process itself. Several large, well-designed longitudinal cohorts provide compelling evidence in favour of the latter hypothesis. Notably, Fang et al. demonstrated that differences in serum TC and LDL-C were already detectable up to 20 years before clinical diagnosis, while Wang et al. observed a further significant decline in cholesterol levels during the prodromal phase and after disease onset. This temporal pattern strongly suggests that the observed hypocholesterolaemia may reflect increased neuronal cholesterol demand for membrane repair, synaptic remodelling and dendritic plasticity, as well as prodromal weight loss and metabolic alterations that precede motor symptoms.

Consequently, low circulating cholesterol might not primarily drive disease risk but could instead represent a secondary phenomenon resulting from the underlying neurodegenerative burden. Although the present systematic review cannot establish definitive causality, the consistent finding of an inverse association between cholesterol levels and PD incidence/progression, combined with the early and progressive decline in lipid concentrations, supports the concept of reverse causation in at least a substantial proportion of cases. Future studies employing Mendelian randomisation approaches and detailed mechanistic investigations will be essential to disentangle causal directions and to determine whether, and in whom, cholesterol modulation might offer neuroprotective potential.

At the biological level, one proposed mechanism to explain this association involves increased neuronal cholesterol demand during prodromal and early stages of PD. Neurons may require more cholesterol for processes such as membrane repair, dendritic remodelling, and synaptic vesicle trafficking. This could lead to enhanced uptake from the periphery, effectively creating a “cholesterol sink” that depletes circulating TC and LDL-C levels, and in turn, may contribute to accelerated neurodegeneration [19]. However, this remains a hypothesis requiring validation through experimental studies.

Further hypotheses relate to brain cholesterol homeostasis. Impaired export of 24-hydroxycholesterol from the brain via reduced activity of enzymes like CYP46A1 may contribute to lysosomal cholesterol accumulation, promoting autophagic dysfunction and α-synuclein aggregation. This effect could be amplified in carriers of mutations in the glucocerebrosidase gene (GBA1), where lysosomal lipid degradation is already compromised [32–35]. In this context, higher peripheral cholesterol levels might indirectly support brain cholesterol supply, potentially mitigating these deficits, though direct causal links remain unestablished.

Cholesterol-rich membrane microdomains (lipid rafts) were also implicated in modulating α-synuclein binding and aggregation [36–38]. Evidence suggests that adequate cholesterol levels stabilize these domains, thereby reducing the propensity for pathological α-synuclein fibrillization and aggregation (positively influencing neuronal stability; e.g. via altered protein confirmation) [39, 40]. Conversely, cholesterol depletion could destabilize rafts and exacerbate aggregation risks [37, 41]. However, oxidative conversion of cholesterol into reactive oxysterols represents a potential counteracting risk: Elevated peripheral cholesterol might increase oxysterol production under oxidative stress, perturbing mitochondrial function and heightening dopaminergic neuron vulnerability [35, 42]. This highlights a delicate balance, where the net neuroprotective effect of higher cholesterol may depend on individual factors like antioxidant capacity [43].

Additionally, as cholesterol is also the precursor for neurosteroids such as pregnenolone and progesterone metabolites, its peripheral depletion could limit the availability of these neuroprotective molecules, further impairing neuronal resilience [44–46]. Overall, these mechanisms underscore the complexity of lipid dynamics in PD and call for targeted studies to disentangle protective versus detrimental pathways.

Dietary factors add yet another dimension. Paradoxically, high adherence to a Mediterranean diet (MD), which modestly lowers TC by about 7.4 mg/dL and LDL-C by a similar margin compared to low-fat regimens, was robustly associated with a 5% reduction in PD risk (OR = 0.75; 5% CI: 0.66–0.84) and slower disease progression [47–49], despite its lipid-lowering effects [50]. In the Swedish cohort of over 47,000 women followed up for more than two decades, the protective association of MD adherence did not appear until after age 65, when each one-point increase in the MD score conferred 29% reduction in PD risk [48]. Similarly, the HELIAD study of 1,731 Greek seniors found that every unit increase in the MD-Score translated toa 2% lower probability of prodromal PD symptoms [47].

Mechanistically, the MD’s rich array of antioxidants (polyphenols such as oleuropein and resveratrol, carotenoids, and vitamins C and E) scavenge reactive oxygen species and inhibit lipid peroxidation, thereby protecting neuronal membranes and preventing α-synuclein misfolding [51, 52]. Its high content of monounsaturated fats (olive oil) and polyunsaturated fats (nuts, fatty fish) enhances HDL-mediated reverse cholesterol transport, safely mobilizing excess neuronal cholesterol and oxysterols (including 24-hydroxycholesterol) out of the brain and averting lysosomal overload [32, 35, 53].

Concurrently, abundant dietary fiber and polyphenols foster butyrate-producing gut bacteria (Faecalibacterium, Roseburia), which reinforce intestinal barrier integrity, lower circulating lipopolysaccharide levels, and suppress systemic inflammation, effects that together support blood-brain barrier function and stabilize central lipid homeostasis [54–56]. In a five-week pilot intervention, PD patients increased their MD score from 4.4 ± 0.6 to 11.9 ± 0.7 (*p* < 0.01), lost an average of 2.5 kg, experienced significant relief from constipation (constipation syndrome score fell from 2.3 ± 0.5 to 1.5 ± 0.3; *p* = 0.04), and showed a decline in pro-inflammatory Bilophila species in their gut microbiome [54].

Given the delayed onset of neuroprotection, often emerging years after dietary implementation, early and sustained MD adherence in midlife likely represents the most effective window for PD prevention [47, 48]. Looking forward, adapted “high-cholesterol” variants of the MD enriched with sterol-dense foods such as eggs, shellfish, and aged cheeses, while preserving the diet’s core antioxidant and prebiotic elements, could hypothetically support central cholesterol availability in at-risk populations, without fully undermining the MD`s established anti-inflammatory and microbiome-mediated benefits [57, 58].

Pharmacological lipid-modifying strategies must also account for the physicochemical diversity of statins. Most commonly prescribed statins (atorvastatin, simvastatin, fluvastatin, lovastatin, pitavastatin) are lipophilic, easily penetrating cell membranes, including extrahepatic tissues and potentially the CNS [59], and are extensively metabolized by CYP3A4 and CYP2C9, which elevates the risk of drug-drug interactions [60].

In contrast, hydrophilic statins (e.g. rosuvastatin, pravastatin) are more hepatoselective: they rely principally on OATP1B1-mediated hepatic uptake, undergo minimal cytochrome P450 metabolism (rosuvastatin only partially via CYP2C9; pravastatin mainly by sulfation), and are largely eliminated unchanged renally, resulting in lower systemic exposure and a reduced potential for CYP-mediated drug-drug interactions [60, 61]. Gastrointestinal pH and renal handling can further influence their bioavailability and excretion, such as hydrophilic molecules may be recovered uncharged in the urine, whereas lipophilic statins (e.g. simvastatin, atorvastatin) are more likely to cross the blood-brain barrier and are typically biotransformed to more polar metabolites prior to renal clearance [59, 62, 63]. These pharmacokinetic distinctions are clinically relevant for PD: if minimizing systemic exposure and interaction risk is the priority, rosuvastatin or pravastatin are reasonable choices; conversely, several observational studies have associated lipophilic statins with lower PD incidence, suggesting possible pleiotropic or neuroprotective effects, though randomized controlled evidence for disease modification is lacking [64, 65].

Therefore, statin selection in patients with or at risk for PD should be individualized, balancing the cardiovascular indication regarding neurological outcomes.

In many epidemiological studies the association between circulating lipids and PD appears sex-dependent: Rozani et al. reported that higher TC/LDL-C over time was associated with lower PD risk predominantly in men, whereas earlier de Lau et al. identified the strongest inverse associations in women, illustrating inconsistent sex-stratified effects across cohorts [23, 27].

Several explanations may account for these discrepancies. First, endogenous oestrogens alter lipid handling – estrogenic signalling up-regulates hepatic LDL-receptor-activity (and modulates PCSK9/LDLR dynamics), which changes LDL clearance and overall lipid profiles and therefore could modify the exposure-disease relationship across the reproductive life course [66, 67].

Second, sex differences in body-fat distribution (visceral versus subcutaneous stores), sex-specific gut-microbiome composition, and sex-hormone-microbiome interactions can plausibly affect systemic lipid metabolism, CNS lipid availability, and neuroinflammatory pathways relevant to PD [68, 69].

Third, gendered factors - e.g., differential healthcare-seeking behaviour, sociocultural dietary patterns, and historical under-representation of women in PD trials and cohort studies - may introduce ascertainment or selection biases that distort observed sex-specific associations [70, 71].

Age and menopausal transition are likely central modifiers: large prospective studies of reproductive-age transitions (e.g. SWAN and related analyses) show that LDL/TC rise and cardiovascular risk increase after menopause, such that any protective or deleterious lipid-PD association that is mediated through lifetime lipid exposure will vary with menopausal status and age [72, 73].

Emerging evidence further implicates cholesterol in PD-related cognitive decline. Jeong et al. reported that higher TC was linked to better executive function in normal-weight PD patients but predicted more rapid cognitive deterioration in obese individuals, highlighting a bidirectional, BMI-modulated effect [31].

Clinically, these observations complicate the notion that universal lipid lowering necessarily confers neuroprotection. A limited number of observational analyses have described a transient association between statin initiation (most notably with lipophilic agents) and an increased rate of early PD diagnoses [74], though this pattern may reflect detection bias, reverse causation, or unmasking of a prodromal phase rather than a direct causal effect. Conversely, prospective cohort and Mendelian-randomization studies that link higher circulating TC and LDL-C with lower PD risk address lipid exposure per se rather than pharmacologic lipid lowering [19, 24]. Dietary guidance should continue to emphasise Mediterranean-type patterns for their broad cardiometabolic and neuroprotective benefits, and targeted research should explore whether modified dietary approaches can preserve central cholesterol availability without compromising cardiovascular risk reduction.

## Limitations

The literature search was restricted to PubMed only. Although PubMed provides extensive coverage of biomedical literature, the possibility of missing relevant studies indexed in other databases cannot be entirely excluded.

While this review provides an overview of the current evidence, several limitations must be acknowledged. Only four studies incorporated clinical scoring systems such as UPDRS or cognitive assessments. These instruments are essential for measuring Parkinson’s disease severity, functional impact, and progression, and their absence may limit the interpretability and generalizability of findings.

In addition, Simon et al. (Table 1) relied solely on self-reported data rather than clinical assessments, introducing the potential for recall bias [24].

Despite these limitations, the overall findings highlight the potential relevance of serum-TC and LDL-C in the context of PD, and underscore the need for further high-quality research to explore their mechanistic and clinical significance.

## Conclusion

In many cohorts, higher peripheral TC and LDL-C are associated with lower incidence of PD, an association most consistent among older men. Other studies, however, suggest that specific lipid fractions such as HDL may exert additional, potentially protective effects. The progressive decline in TC levels before and after PD diagnosis supports the hypothesis that lipid dysregulation may both reflect and influence neurodegenerative processes, possibly through altered neurosteroid availability and compromised neuronal resilience. Early TC reduction may contribute to the observed inversed relationship with PD risk, emphasizing the dynamic nature of lipid metabolism and the need for further research to substantiate these proposed pathways.

This systematic review demonstrates a consistent inverse association between peripheral total cholesterol and LDL-C levels and both the incidence and progression of Parkinson’s disease across multiple large longitudinal cohorts. Most included studies were of good methodological quality (7–9 stars on the Newcastle-Ottawa Scale).

Importantly, several lines of evidence suggest that lower peripheral cholesterol levels may be, at least partly, a consequence rather than the primary cause of PD. Longitudinal data show a decline in cholesterol already during the prodromal phase (up to 20 years before diagnosis) and a further reduction after clinical manifestation. These findings are consistent with increased neuronal cholesterol demand, weight loss, and metabolic alterations in prodromal and manifest PD. Nevertheless, residual confounding and reverse causation cannot be fully excluded. Future studies employing Mendelian randomisation approaches and detailed mechanistic investigations are needed to clarify the causal relationship.

Given the complex interplay between peripheral lipid metabolism, confounding factors (such as statin use, BMI and diet), and disease progression, simple lipid metrics alone are currently insufficient to guide neuroprotective strategies in PD. However, a deeper understanding of lipid dynamics in PD may open new avenues for biomarker development and personalised therapeutic approaches in the future.

These heterogeneous findings, together with signals of a transient rise in early PD diagnoses shortly after statin initiation in some observational datasets, underscore that simple lipid metrics (e.g. a single TC or LDL value) are insufficient to characterise PD risk or to direct neuroprotective strategies. Choosing a hydrophilic statin (e.g. rosuvastatin, pravastatin) to minimise central nervous system exposure may become a reasonable option. Nevertheless, until now this strategy is not supported by enough evidence from clinical trials to change prescriptions practice. Any preferential recommendation should, however, be graded and caveated because randomized evidence for differential neurological outcomes is lacking.

Finally, the apparent paradox between higher peripheral cholesterol being associated with lower PD risk while cholesterol-lowering diets improve many neurocognitive outcomes highlights the need for mechanistic and longitudinal data that reconcile peripheral metabolic health with central cholesterol handling.

## Electronic Supplementary Material

Below is the link to the electronic supplementary material.

Supplementary Material 1

## Data Availability

Not applicable. No datasets were generated or analysed during the current study.
