# Supplementary material for: When bad turns good: a systematic review on cholesterol and LDL in longitudinal patient cohorts with Parkinson’s disease
Source: Neurol Sci. 2026 May 13;47(6):491. doi: 10.1007/s10072-026-09070-9 (PMC13171655; doi:10.1007/s10072-026-09070-9)
Supplement: Supplementary file 1 — Supplementary Material 1 [file 10072_2026_9070_MOESM1_ESM.docx]

Supplementary Table 1 Quality assessment of included studies using the Newcastle-Ottawa Scale (NOS) for cohort studies

| **Nr.** | **Study** | **Year** | Selection (max 4) | Comparability (max 2) | Outcome (max 3) | Total (max 9) | **Quality** |
| --- | --- | --- | --- | --- | --- | --- | --- |
| 1. | De Lau et al. | **2006** | ******** | ****** | ******* | **9** | **Good** |
| 2. | Simon et al. | **2007** | ******** | ***** | ******* | **8** | **Good** |
| 3. | Huang et al. | **2008** | ******** | ****** | ******* | **9** | **Good** |
| 4. | Hu et al. | **2008** | ******** | ***** | ******* | **8** | **Good** |
| 5. | Huang et al. | **2011** | ******** | ****** | ******* | **9** | **Good** |
| 6. | Huang et al. | **2015** | ******** | ****** | ******* | **9** | **Good** |
| 7. | Sterling et al. | **2016** | ******** | ****** | ******* | **9** | **Good** |
| 8. | Rozani et al. | **2018** | ******** | ****** | ******* | **9** | **Good** |
| 9. | Fang et al. | **2019** | ******** | ****** | ******* | **9** | **Good** |
| 10. | Huang et al. | **2019** | ******** | ***** | ******* | **8** | **Good** |
| 11. | Yokoi et al. | **2020** | ******* | ***** | ******* | **7** | **Good** |
| 12. | Wang et al. | **2021** | ******** | ****** | ******* | **9** | **Good** |
| 13. | Hurh et al. | **2022** | ******** | ****** | ******* | **9** | **Good** |
| 14. | Jeong et al. | **2024** | ******** | ****** | ******* | **9** | **Good** |

**Note:**
Quality was assessed using the Newcastle-Ottawa Scale (NOS) for cohort studies [74]. Most studies achieved good quality (7-9 stars). Comparability was occasionally limited due to incomplete adjustment for confounders such as statin use or BMI.
